# A novel eco-friendly HPTLC approach for the simultaneous determination of erdosteine, ibuprofen and pseudoephedrine pharmaceutical combination

**DOI:** 10.1038/s41598-026-57106-z

**Published:** 2026-06-16

**Authors:** Feda A. H. Elgammal, Maram G. Hafez, Hadeel A. Khalil, Dina A. Gawad, Tarek S. Belal

**Affiliations:** 1 Pharmaceutical Analytical Chemistry Department, Faculty of Pharmacy, University of Alexandria, Elmessalah, Alexandria, 21521 Egypt; 2 Postgraduate Student, Faculty of Pharmacy, University of Alexandria, Elmessalah, Alexandria, 21521 Egypt

**Keywords:** Erdosteine, Ibuprofen, Pseudoephedrine, HPTLC, Greenness assessment, Sustainability assessment, Chemistry, Drug discovery

## Abstract

This paper introduces the first validated and ecofriendly high-performance thin-layer chromatographic (HPTLC) method for the simultaneous determination of erdosteine (ERD), ibuprofen (IBU), and pseudoephedrine (PSE) pharmaceutical combination. This drug combination aids in treating symptoms associated with upper respiratory tract infections (URTIs) by providing mucolytic, analgesic, anti-inflammatory, and decongestant effects within a single therapeutic regimen. The chromatographic separation was effectively achieved on a silica gel 60F_254_ plate using a mobile phase composed of ethyl acetate: methanol: ammonia (7:1.4:0.5, v/v), and UV detection at 210 nm. The analytes were well resolved with retardation factors (R_f_) of 0.06, 0.23, and 0.35 for ERD, IBU, and PSE, respectively. The method demonstrated high accuracy, with recoveries ranging from 98.4% to 101.2% and excellent precision, as indicated by % RSD values below 2%. It also showed high sensitivity, with detection limits of 0.014 µg/spot, 0.002 µg/spot and 0.150 µg/spot for ERD, IBU, and PSE, respectively. The method’s environmental sustainability was confirmed using the Analytical EcoScale, Analytical GREEnness (AGREE) calculator and Green Analytical Procedure Index (GAPI). Additionally, its overall whiteness profile was assessed using the Red-Green-Blue (RGB12) model approach, highlighting its analytical efficiency and eco-friendly design.

**Supplementary Information:**

The online version contains supplementary material available at 10.1038/s41598-026-57106-z.

## Introduction

Upper respiratory tract infections (URTIs) are widespread conditions affecting the nose, throat, and airways. They are most often viral in origin, although bacterial causes may also occur. URTIs include conditions like the common cold, sinusitis, laryngitis, and pharyngitis^1^. Most URTIs are mild and self-resolved within a week or two with the help of the body’s immune system. As a result, treatment primarily focuses on relieving the symptoms associated with URTIs to improve comfort and quality of life during the recovery period^2^.

Nasal congestion is one of the most common symptoms associated with URTIs. It results from inflammation of the blood vessels in the nasal passages, causing swelling of the nasal tissues. This inflammatory response causes a feeling of stuffiness or blockage in the nose, making it difficult to breathe through the nostrils. In many cases, congestion is accompanied by an increase in mucus production, which can further obstruct the nasal passages and lead to a runny nose^3^.

**Fig. 1:**
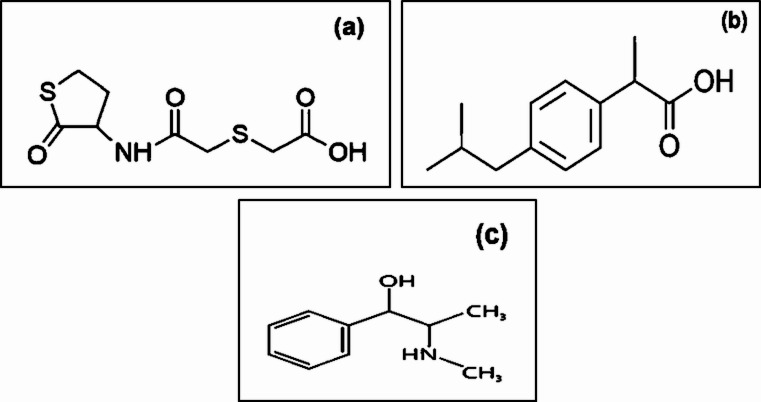
Structural formula of **(a)** ERD, **(b)** IBU and **(c)** PSE.

Erdosteine (ERD), featuring a carboxyl group and a thiolactone ring, Fig. 1a, is a powerful mucolytic and mucoregulatory agent^4,5^. It controls mucus production, reduces viscosity, and improves mucociliary transport, resulting in faster expectoration and mucolytic actions^6^. When taken orally, ERD is rapidly transformed into its active metabolite^7^. Its two sulfhydryl groups provide free radical scavenging and antioxidant properties^4,5,8^. Besides its mucolytic benefits, ERD also has antitussive effects^4,9^. The recommended oral dose of ERD is 300 mg twice a day^10^. Ibuprofen (IBU), a derivative of propionic acid, Fig. 1b, is a highly effective non-steroidal anti-inflammatory drug (NSAID) with well-established analgesic, and antipyretic properties^11^. At low doses, IBU is as effective as aspirin and more effective than the equivalent dose of paracetamol^12^. The mechanism of action of IBU involves blockade of cyclooxygenase enzymes (COX-1 and COX-2), thereby inhibiting the production of prostaglandins responsible for inflammation, pain, and fever^13^. Due to its short plasma half-life, IBU has a relatively short action time of 1 to 2 h and requires to be administered 3 to 6 times a day^14^. The recommended oral daily dose ranges from 1.2 to 1.8 g, divided into multiple doses^10^. Pseudoephedrine (PSE), Fig. 1c, exhibits both direct and indirect sympathomimetic decongestant effects. It is rapidly and fully absorbed, with maximum plasma levels reached within1 to 4 h after oral intake^15^. Typically, PSE is administered orally at a dose of 60 mg, 3 to 4 times a day^10^.

Combining these three drugs in a single-dose formulation for the symptomatic treatment of URTIs is a promising approach. This combination leverages the decongestant properties of PSE, the anti-inflammatory effects of IBU, and the mucolytic action of ERD to provide comprehensive relief from multiple symptoms of URTIs. Additionally, combining multiple active pharmaceutical ingredients (APIs) in a single formulation simplifies the medication regimen, reducing the number of pills needed. This approach can improve patient compliance, enhance therapeutic effects, reduce resistance risk, and provide convenience and cost-effectiveness.

Literature review revealed numerous analytical methods for determining ERD, IBU, and PSE, either alone or in different combinations, including HPLC^16–24^, electrochemical^25–28^, spectrophotometric^29–35^, spectrofluorimetric^36–41^, gas chromatographic^42–44^, and HPTLC^45–49^ methods. Only a single analytical report could be retrieved for quantitation of this triple combination using reversed phase HPLC. The 3 drugs were separated using C18 column operated at 40 °C and a gradient elution system consisting of sodium formate (pH 3.2), deionized water and methanol for a total run time of 18 min^50^. Up to date, there is no reported HPTLC method for the simultaneous analysis of these three drugs. HPTLC offers multiple benefits compared to HPLC and therefore is occasionally preferred over it. HPTLC uses significantly less solvents and sample volumes, making it more cost-effective and ecologically friendly. Moreover, it is a quick analytical approach that enables the concurrent handling of a significant number of samples in parallel with minimum energy and solvent consumption. In fact, HPTLC does not need complex procedures or the advanced instrumentation typically related to HPLC analysis methods^51–55^.

The proposed HPTLC analytical method is not just environmentally friendly as proven by AGREE, analytical Eco-scale and GAPI approaches, but also the combination of these three drugs in a single formulation reduces the ecological impact. It reduces the overall usage and disposal of medications. This is crucial since unused medications can persist in the environment and cause long-term ecological damage. In addition, to ensure the analytical performance of the suggested HPTLC method alongside its environmental aptness, the Red-Green-Blue (RGB12) model approach was performed for sustainability and whiteness assessment.

## Experimental

### Instrumentation

HPTLC experiments were achieved using a CAMAG microliter syringe (100 µL) in conjunction with a CAMAG Linomat IV automated sample applicator (Switzerland), under a nitrogen stream. The mobile phase was developed in the ascending mode using a CAMAG twin-trough glass chamber (15 cm x 20 cm x 30 cm) (Switzerland). Densitometric measurements were conducted using CAMAG TLC Scanner III, equipped with a deuterium lamp that emits continuous UV radiation ranging from 190 to 400 nm. The TLC scanner was operated via CAMAG TLC software (CATS, version 3.15).

### Sample application and chromatographic development

Aliquots of 10 µL were applied as bands of 5 mm width, separated by 5 mm, at a distance of 15 mm from the bottom edge and 10 mm from each side of the plate. Each sample is applied in duplicate and air-dried for 5 min. Prior to development, the TLC chamber was pre-saturated for 20 min at room temperature with the mobile phase (ethyl acetate: methanol: ammonia in a ratio of 7:1.4:0.5, v/v) to ensure uniform solvent migration. Following development, spectro-densitometric detection of the resolved components was conducted at 210 nm.

### Reagents and materials

IBU and PSE were provided by Pharco Pharmaceuticals Co., Alexandria, Egypt. ERD was obtained from Borg Pharmaceutical Industries, located in New Borg El-Arab City, Alexandria, Egypt. Reagents used in the study included 30% ammonia solution (Loba Chemie Ltd., Mumbai, India), ethyl acetate (S.D. Fine Chem Ltd., Mumbai, India), and methanol of HPLC-grade (Fisher Scientific, Loughborough, UK). All compounds were of high purity and appropriate analytical quality. The studied drugs were obtained with purity ≥ 99%, while all other chemicals and reagents were of analytical grade and used without further purification. Precoated HPTLC aluminum plates (20 × 20 cm) with a 250 μm layer of silica gel 60 F_254_) were obtained from E. Merck (Darmstadt, Germany).

### Stock and working solutions preparation

IBU and ERD stock solutions (1 mg/mL) were made up by dissolving 10 mg of each analyte separately in methanol and diluting to volume in 10 mL volumetric flasks. Similarly, PSE stock solution (4 mg/mL) was obtained by dissolving 40 mg in 10 mL methanol. All stock solutions were freshly prepared daily to ensure stability and reliability of the results. Further dilutions using methanol as solvent were performed to obtain working solutions within the concentration ranges of 8–200 µg/mL for ERD, 5–200 µg/mL for IBU, and 80–1000 µg/mL for PSE.

### Construction of the calibration plots

Aliquots of 10 µL from each analyte working solution were spotted onto TLC plates to get the final concentrations of 0.08–2 µg/spot for ERD, 0.05–2 µg/spot for IBU, and 0.8–10 µg/spot for PSE. Sample application, plate development, and scanning were performed as formerly described. Calibration curves were constructed by plotting the measured peak areas versus their respective concentrations. Linear regression analysis was employed to define the relationship between concentration and response.

### Analysis of laboratory-prepared mixtures

Synthetic mixtures of ERD, IBU, and PSE were prepared in triplicates at concentrations reflecting those in marketed products by combining measured aliquots from their corresponding standard solutions into 10 mL volumetric flasks and the volume was completed with methanol. The first mixture was prepared to yield final concentrations of 150 µg/mL ERD, 200 µg/mL IBU, and 30 µg/mL PSE, where ERD and IBU concentrations fell within their established calibration ranges for quantification. The second mixture was prepared at higher concentrations of 750 µg/mL ERD, 1000 µg/mL IBU, and 300 µg/mL PSE to ensure reliable quantification of PSE within its calibration range. Both mixtures were analyzed under the chromatographic conditions previously depicted.

## Results and discussion

### Method development and optimization

The primary objective in developing and optimizing the proposed HPTLC method was to obtain symmetrical, well-resolved peaks with reproducible and acceptable R_f_ values. A systematic series of trials was conducted on the mobile phase composition for effective chromatographic separation of ERD, IBU, and PSE.

Initial attempts employed a mobile phase composed of ethyl acetate, acetone, and ammonia in a ratio of (2.5:2.5:0.5, v/v). This system proved ineffective, as PSE migrated to the solvent front while ERD remained at the starting line. Adjusting the ratios by increasing ethyl acetate or acetone individually failed to improve the separation. Next, ammonia in this system was replaced by acetic acid while keeping the same mobile phase ratio. This switch led both PSE and ERD to migrate to the solvent front. Although increasing acetone reduced the R_f_ of PSE, ERD and IBU, however, it exhibited high R_f_ values with no significant separation.

A different approach using dichloromethane, methanol, and acetic acid (5:2.5:0.5, v/v) resulted in ERD and IBU migrating to the solvent front. Increasing dichloromethane lowered their R_f_ values but failed to separate them. Additional trials with various combinations such as (acetone, ether, and ammonia), (ethanol and water), (butanol and acetic acid), (acetonitrile, ethanol, and acetic acid), (ethanol, ether, and water), (ether and ammonia), (ether and acetic acid), and (propranolol, ether, and acetic acid) did not yield satisfactory separation.

Further trial with ethyl acetate, methanol, and acetic acid (5:2.5:0.5, v/v) showed no separation between ERD and IBU. Ether was introduced into the system, resulting in a modified solvent composition of ethyl acetate, methanol, ether, and acetic acid (5:2.5:2.5:0.5, v/v). However, the addition of ether had no significant impact on the separation, and the analytes remained unresolved. A simpler mixture of ethyl acetate, ether, and acetic acid (5:2.5:0.5, v/v) resulted in a very low R_f_ for PSE, with no improvement in the separation of ERD and IBU, even after increasing ethyl acetate. To reduce polarity, hexane was incorporated into the mobile phase, and it caused IBU migration to the solvent front without improving separation. On the other hand, a mixture of ethyl acetate, hexane, and acetic acid (4:7.5:2, v/v) caused PSE to remain at the starting line and ERD to show very low R_f_. To enhance resolution, methanol was added to the system, yielding a modified composition of ethyl acetate, hexane, methanol, and acetic acid (2:5:1:0.5, v/v). Although this adjustment improved separation, it raised concerns due to hexane’s environmental impact.

**Fig. 2:**
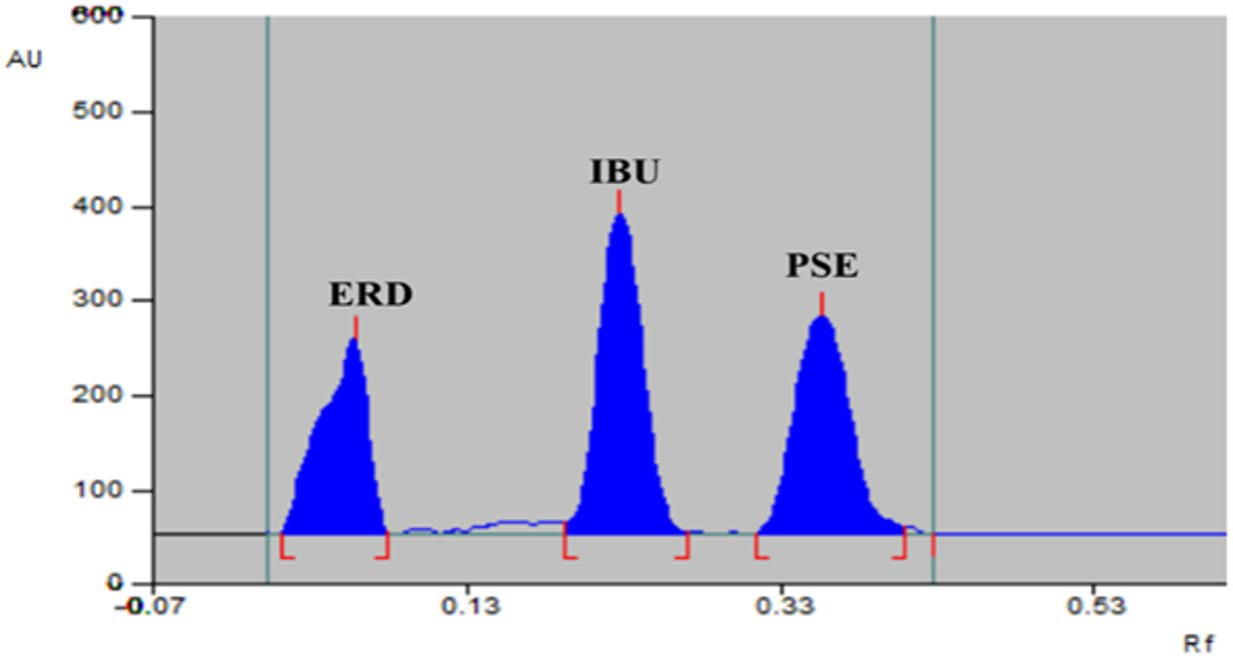
HPTLC densitogram of a mixture containing 2 µg/spot ERD, 2 µg/spot IBU and 10 µg/spot PSE at R_f_ values of 0.06, 0.23 and 0.35, respectively.

An alternative system using ethyl acetate, ether, methanol, and ammonia (10:3:6:0.5, v/v) provided good separation but was considered overly complex. Ether was eliminated, and the mobile phase was simplified to ethyl acetate, methanol, and ammonia (5.5:2.5:0.5, v/v). A good resolution between the three analytes was finally achieved. Further optimization was carried out by fine-tuning the ratio by increasing ethyl acetate and decreasing methanol. The ultimate optimized mobile phase is composed of ethyl acetate, methanol, and ammonia (7.5:1.4:0.5, v/v). This mobile phase combination enabled clear separation of ERD, IBU, and PSE with R_f_ values of 0.06 ± 0.02, 0.23 ± 0.03, and 0.35 ± 0.02, respectively, (Fig. 2). System suitability parameters including resolution and tailing factors were calculated and found to be within acceptable limits, confirming the reliability and aptness of the proposed HPTLC method (Table 1S in the Supplementary File.).

Other Chromatographic parameters were carefully evaluated and optimized to ensure efficient separation of the target analytes including band application, and detection wavelengths. To optimize sample application parameters, various band widths (4, 5, and 6 mm) and inter-band spacings (4, 5, and 6 mm) were systematically evaluated to achieve a balance between peak symmetry and maximum sample capacity per plate. The optimal configuration of 5 mm bandwidth with 5 mm interspace permitted the spotting of up to 18 bands on a single 10 × 20 cm TLC plate. This setup provided well-defined, symmetrical peaks with satisfactory sharpness, ensuring both analytical efficiency and spatial utilization.

Densitometric scanning of the developed plate was performed across the 200–400 nm range to determine the optimum wavelength for simultaneous measurement of ERD, IBU, and PSE. Based on the UV-absorption spectra obtained (Fig. 1S, supplementary file), 210 nm was selected for quantification of all three analytes in a single analysis.

### Method validation

Validation of the proposed HPTLC method was conducted in accordance with the International Council for Harmonisation (ICH) guidelines (Q2 (R1))^56^.

#### Linearity and analytical concentration ranges

Linearity was evaluated by analyzing a minimum of 5 concentration levels for each drug applied in duplicates onto HPTLC plate. The concentration ranges were 0.08–2 µg/spot for ERD, 0.05–2 µg/spot for IBU, and 0.8–10 µg/spot for PSE. Calibration curves were established by plotting the chromatographic peak areas versus their corresponding analyte concentrations (Fig. 2S, supplementary file). Table 1 summarizes the analytical performance and statistical parameters for the proposed method. Regression analysis confirmed a strong linear relationship across all tested concentrations, as indicated by good correlation coefficients (*r* ≥ 0.999) and RSD of the slope (S_b_%) not exceeding 2%.

**Table 1:** Analytical parameters for the determination of ERD, IBU and PSE using the proposed HPTLC method.

Parameters	ERD	IBU	PSE
Wavelength (nm)	210	210	210
R_f_	0.06 ± 0.02	0.23 ± 0.03	0.35 ± 0.02
Concentration range (µg/spot) (equivalent to µg/mL calibration standards)*	0.08–2 (8-200)	0.05–2 (5–200)	0.8–10 (80–1000)
Intercept (a),	81.03	590.71	362.26
Standard deviation of the intercept (S_a_)	26.99	33.43	24.40
Slope (b),	1610.60	3427.71	351.16
Standard deviation of the slope (S_b_)	29.94	34.14	5.42
% RSD of the slope	1.86	1.00	1.54
Correlation coefficient (r)	0.9993	0.9997	0.9995
Standard deviation of residuals (S_y/x_)	47.90	63.78	44.95
LOD (µg/spot)	0.014	0.002	0.150
LOQ (µg/spot)	0.048	0.008	0.500

* Spotting volume = 10 µL.

#### Accuracy and precision

To evaluate accuracy and precision of the developed method, three replicates at each of three selected concentration levels of ERD, IBU, and PSE standards were analyzed within a single day (intra-day repeatability) and over three consecutive days (inter-day precision). As presented in Table 2, the percentages relative standard deviation (%RSD) and relative error (%Er) values for both intra-day and inter-day assessments were below 2%, demonstrating the method’s excellent accuracy and precision.

**Table 2:** Accuracy and precision of the proposed HPTLC method.

Analyte	Concentration (µg/spot)	Intra-day Precision (Repeatability)			Inter-day (Intermediate) Precision		
		Found ± SD^a^ (µg/spot)	RSD (%)	Er (%)	Found ± SD^a^ (µg/spot)	RSD (%)	Er (%)
ERD	0.1	0.1012 ± 0.0015	1.48	1.20	0.0984 ± 0.0015	1.52	-1.60
	0.7	0.7027 ± 0.0120	1.71	0.39	0.7018 ± 0.0018	0.26	0.26
	2.0	2.0013 ± 0.0182	0.91	0.07	1.9982 ± 0.0050	0.25	-0.09
IBU	0.05	0.0506 ± 0.0004	0.79	1.20	0.0499 ± 0.0002	0.40	-0.20
	0.7	0.7023 ± 0.0083	1.18	0.33	0.7012 ± 0.0026	0.37	0.17
	2.0	2.0103 ± 0.0320	1.59	0.52	1.9999 ± 0.0056	0.28	-0.005
PSE	2	2.0135 ± 0.0295	1.47	0.68	2.0082 ± 0.0360	1.79	0.41
	7	6.9721 ± 0.0998	1.43	-0.40	6.9763 ± 0.1272	1.82	-0.34
	10	10.0569 ± 0.0699	0.70	0.57	9.9297 ± 0.0816	0.82	-0.70

^a^ Mean ± Standard deviation of three replicates.

#### Detection limit (LOD) and quantitation limit (LOQ)

According to ICH guidelines, LOD and LOQ were assessed using signal-to-noise ratio of 3:1 and 10:1, respectively^56^. As presented in Table 1, the proposed method shows LOD as low as 0.014 µg/spot for ERD, 0.002 µg/spot for IBU, and 0.150 µg/spot for PSE. The LOQ was determined to be 0.048 µg/spot for ERD, 0.008 µg/spot for IBU, and 0.500 µg/spot for PSE.

#### Selectivity and specificity

To assess the method’s selectivity, laboratory-prepared mixtures of the three analytes were analyzed at different concentrations within their established linearity ranges, and the concentrations of each component were determined based on their corresponding calibration regression equations. The recovered concentrations, %RSD; and %Er presented in Table 3 were found to be satisfactory, thereby confirming the method’s selectivity and its ability to accurately resolve and quantify ERD, IBU, and PSE across different ratios.

**Table 3:** Determination of ERD – IBU – PSE laboratory-prepared mixtures using the proposed HPTLC method.

Concentration (µg/spot)			ERD			IBU				PSE		
ERD	IBU	PSE	Found ± SD^a^ (µg/spot)	RSD (%)	Er (%)	Found ± SD^a^ (µg/spot)		RSD (%)	Er (%)	Found ± SD^a^ (µg/spot)	RSD (%)	Er (%)
0.4	0.4	4	0.41 ± 0.006	1.36	1.96	0.39 ± 0.005	1.36		-1.89	3.95 ± 0.034	0.87	-1.21
0.6	0.6	6	0.59 ± 0.007	1.23	-1.71	0.61 ± 0.007	1.14		1.64	5.92 ± 0.058	0.98	-1.37
2	2	10	2.03 ± 0.02	0.99	1.41	1.99 ± 0.008	0.42		-0.58	9.95 ± 0.033	0.34	-0.47

^a^ Mean ± Standard deviation of three replicates.

**Fig. 3:**
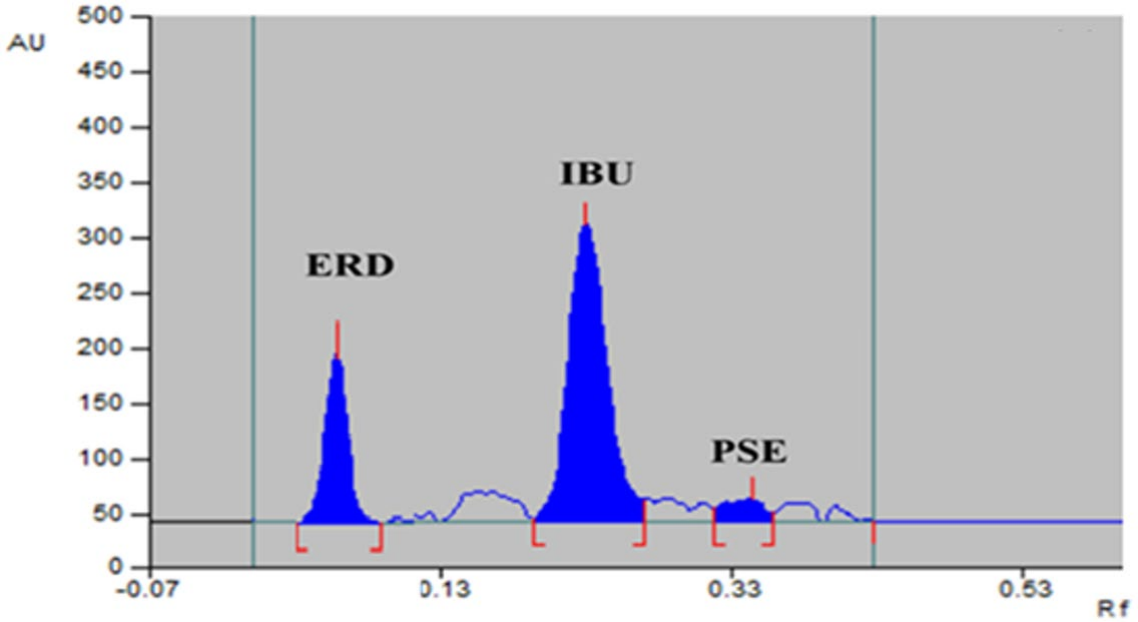
A representative HPTLC densitogram illustrating the separation of the three drugs in the laboratory-prepared mixture.

Moreover, method’s specificity was evaluated by assessing the peak purity of ERD, IBU, and PSE in mixture solutions. This was confirmed through overlaid UV spectra obtained at various time points during peak elution. The corresponding peak purity spectra are presented in Fig. 3S, supplementary file.

#### Robustness

The robustness of the developed HPTLC method was examined by applying deliberate, small variations to the optimized parameters, one at a time. These included slight variation in the proportions of ethyl acetate and methanol in the mobile phase composition (± 0.1%) and volume (± 0.5%), and scan wavelength (± 1 nm). The impact of these changes was assessed using replicate applications (*n* = 3) of a standard mixture composed of 2 µg/spot ERD, and 2 µg/spot IBU and 5 µg/spot PSE. The changes observed in peak area and R_f_ values were statistically insignificant, confirming the method’s reliability and robustness for routine simultaneous determination of ERD, IBU, and PSE.

### Application: Analysis of laboratory-prepared mixtures

As no commercially available dosage form combining the three drugs exists, synthetic mixtures of ERD, IBU, and PSE were successfully prepared at concentrations reflecting typical therapeutic levels. The selected concentrations were based on those found in marketed products, specifically Muctotec capsules^®^ (150 mg ERD), Sinlerg tablets^®^ (200 mg IBU and 30 mg PSE) and Anti-flu capsules^®^ (200 mg IBU and 60 mg PSE).

Two mixtures were accurately quantified under the conditions previously described in the Experimental section. The first mixture was 1.5 µg/spot ERD, 2 µg/spot IBU, and 0.3 µg/spot PSE, where ERD and IBU concentrations fell within their linear ranges for quantification. The second mixture was analyzed using higher concentrations of 7.5 µg/spot ERD, 10 µg/spot IBU, and 3 µg/spot PSE, aligning with the defined linear range for PSE to be quantified.

The chromatographic profiles of both mixtures exhibited well-resolved peaks with no significant interference or co-elution. Additionally, the data listed in Table 4 demonstrated high accuracy and precision, with RSD below 2% and minimal %Er, confirming the method’s reliability for simultaneous determination of the three compounds in combined formulations. A representative densitogram illustrating the resolution of the three drugs in the laboratory-prepared mixture is presented in Fig. 3.

**Table 4:** Application of the proposed method to the analysis of ERD-IBU-PSE laboratory-prepared mixtures based on concentrations found in marketed products.

Nominal value (µg/spot)			Found ± SD^a^ (µg/spot)				
ERD	IBU	PSE	ERD	IBU	PSE	RSD (%)	Er (%)
**1.5**	2	0.3	1.5428 ± 0.0302			1.96	2.853
1.5	**2**	0.3		2.0017 ± 0.0012		0.06	0.085
7.5	10	**3**			2.9725 ± 0.0195	0.66	-0.917

^a^ Mean ± Standard deviation of three replicates.

### Greenness profile assessment

As the demand for environmentally sustainable practices in analytical chemistry grows, evaluating the greenness of the developed analytical methods has become increasingly important. Three prominent tools were employed for this purpose: Analytical Eco-Scale, Analytical GREEnness (AGREE) calculator and Green Analytical Procedure Index (GAPI). These tools facilitated a comprehensive comparison between the developed method and previously reported chromatographic techniques including an HPTLC method for the individual analysis of ERD^47^, another HPTLC method for IBU and PSE mixture^48^; an HPLC method for analyzing a mixture of the three drugs^50^; and finally HPLC method targeting the binary mixture of IBU and PSE^22^.

Analytical Eco-scale^57^ is a quantitative assessment tool that assigns an initial score of 100 and reduces it by applying penalty points (PPs) based on environmental and safety impacts of different items in the procedure. This eco-scale score serves as an indicator of method greenness; with higher values reflecting superior environmental performance. Table 5 demonstrated the PPs assigned to various parameters including the quantity and type of solvents and reagents used, instrument energy requirements, occupational safety considerations, and waste output. The proposed method showed the highest Eco-score, indicating superior environmental friendliness in comparison with the other reported chromatographic methods.

**Table 5:**
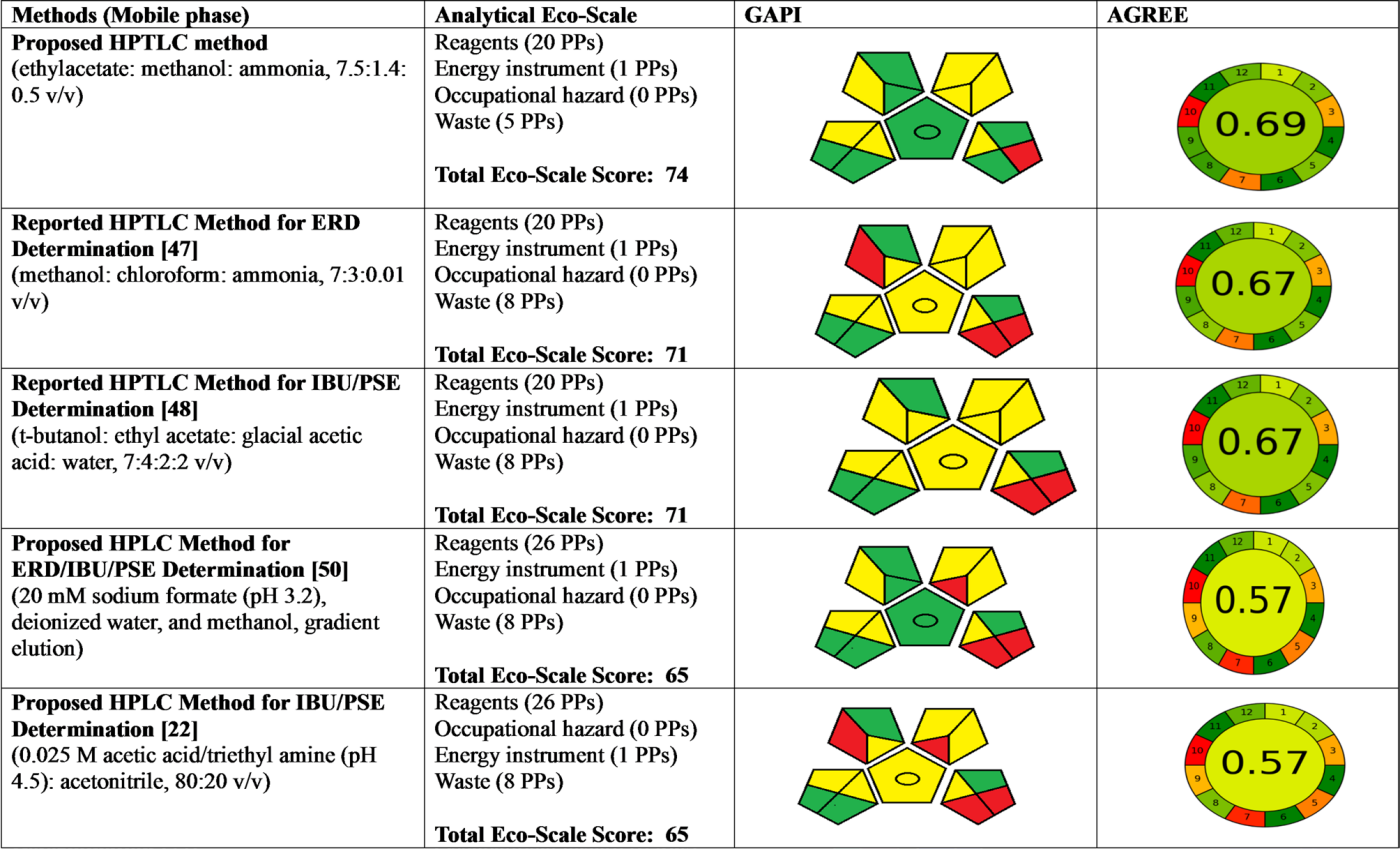
Greenness assessment and comparison with other reported methods.

GAPI approach^58^ offers a visual and comprehensive assessment of the entire HPTLC workflow, from sample collection and preparation to chromatographic separation and detection. It uses a color-coded pictogram to highlight the environmental impact level of each step: green denotes low impact, yellow indicates moderate impact, and red highlights high environmental concern. GAPI pictograms, shown in Table 5, are generated based on the data summarized in Table 2S, supplementary file. the results clearly demonstrate that the developed HPTLC method exhibits the least environmental risk relative to the other reported methods.

The final greenness evaluation was conducted using AGREE software^59^. This assessment criterion is based on the 12 green analytical chemistry (GAC) principles (SIGNIFICANCE). Each principle is assigned a score from 0 to 1, and the software automatically generates a visual output in the form of a clock-like graph. In this graph, each segment represents one of the GAC principles, while the overall greenness score is displayed at the center, accompanied by a color-coded indicator. A dark green indicates excellent adherence to the GAC principles, whereas lighter shades of green indicate adequate but not perfect adherence. Yellow suggests moderate adherence, and red highlights significant areas where the method is not environmentally friendly. According to the AGREE assessment, the proposed method outperforms previously reported approaches, achieving a score of 0.69, as shown in Table 5.

### Whiteness profile assessment

The greenness assessment of the developed method was initially conducted to evaluate its environmental sustainability. However, environmental impact alone does not fully reflect the functional performance of an analytical method. For this reason, the multicriteria RGB 12 model approach was applied to assess the whiteness of the developed method. The term ‘‘whiteness’’ is derived from the combination of the three primary colors (red, green, and blue), together produce white. Similarly, the RGB 12 model integrates three main criteria which are the environmental sustainability (represented by green), analytical performance (represented by red) and economic aspects (represented by blue). When a method satisfies all three criteria, it is considered “white” indicating a balanced and optimal analytical approach. The whiteness profile of the current new HPTLC platform was compared with the previously reported chromatographic procedures^22,47,48,50^.

The developed HPTLC method demonstrated a high red score (97.5%), which is comparable to the HPLC method used for the simultaneous analysis of this ternary mixture (98.8%)^50^. This strong performance indicates the method’s excellent accuracy, precision, and robustness in quantifying all three drugs effectively. It demonstrates that HPTLC, when properly optimized, can rival HPLC in analytical capability. In terms of environmental impact, the proposed method recorded the highest green score of 93.8% among all evaluated techniques. This is attributed to its limited consumption of organic solvents, lower waste output, and the use of eco-friendly reagents. Compared to HPLC methods, which typically involve higher solvent consumption and energy usage, HPTLC offers a significantly greener alternative. All HPTLC-based methods, including the proposed one, attained a blue-category score of 93.3%, reflecting superior cost-efficiency. This is largely due to the lower operational costs, simpler instrumentation, and reduced resource requirements associated with HPTLC. In contrast, HPLC methods showed lower blue scores (ranging from 82.1% to 83.3%), reflecting the higher costs of equipment, maintenance, and consumables.

By averaging the red, blue and green scores, the developed HPTLC method yielded an overall whiteness score of 94.9%, clearly demonstrating its balanced excellence across all three dimensions. This positions it as a truly “white” method under the RGB 12 model, combining high analytical performance with compelling environmental and economic merits. In comparison, while HPLC methods may offer slightly higher analytical precision, they fall short in sustainability and cost-effectiveness. The proposed HPTLC method thus represents a more holistic and practical solution for routine pharmaceutical analysis. These RGB 12 evaluation results of the proposed method alongside other reported chromatographic techniques are illustrated in Table 6.

**Table 6:** RGB model evaluation of the developed method compared with other reported chromatographic methods.

Method	Redness (%)	Greenness (%)	Blueness (%)	Whiteness (%)
Proposed HPTLC	97.5	93.8	93.3	94.9
HPTLC (ERD)^47^	85.0	91.3	93.3	89.9
HPTLC (IBU/PSE)^48^	87.5	91.3	93.3	90.7
HPLC (ERD/IBU/PSE)^50^	98.8	87.5	83.3	89.9
HPLC (IBU/PSE)^22^	87.5	87.5	82.1	85.7

### Comparison with relevant reported methods

Different analytical approaches have been documented for the individual or binary analysis of ERD, IBU, and PSE. Among these techniques, chromatographic methods, particularly HPTLC, stand out for their superior accuracy, specificity, and selectivity compared to traditional spectrophotometric approaches, making them more suitable for complex pharmaceutical formulations.

Several HPTLC methods have been developed for the selected compounds, though none address the simultaneous analysis of ERD, IBU, and PSE as a ternary mixture. Shah et al. established an HPTLC method based on Box-Behnken design for the simultaneous determination of ERD, guaiphenesin, and terbutaline sulphate in syrup, employing a mobile phase of toluene–dichloromethane–methanol–glacial acetic acid (4:4:1.81:0.2, v/v) with detection at 225 nm^46^. Mhaske & Dhaneshwar introduced a stability-indicating HPTLC method for ERD using toluene–methanol–acetone–ammonia (3.5:3.5:2.5:0.05, v/v) and performing densitometric analysis at 254 nm^49^. Moreover, Mostafa et al. presented another stability-indicating HPTLC method for ERD using methanol–chloroform–ammonia (7:3:0.01, v/v) with UV scanning at 235 nm^47^. These methods, while analytically sound, rely on hazardous solvents such as dichloromethane, toluene, and chloroform, which are known for their toxicity, volatility, and environmental persistence.

In contrast, other reported methods have adopted safer solvent systems. Chitlange et al. developed an HPTLC method for the simultaneous determination of IBU and PSE in tablets using a mobile phase of t-butanol–ethyl acetate–glacial acetic acid–water (7:4:2:2, v/v) with analysis carried out at 254 nm^48^. In addition, Moustafa et al. introduced an HPTLC method for the simultaneous analysis of chlorpheniramine, PSE, and IBU in tablets using ethyl acetate–methanol–ammonia (8:2:0.8, v/v) and UV detection at 262 nm, offering relatively safer alternatives^45^.

To date, the simultaneous analysis of ERD, IBU, and PSE has only been reported using HPLC, as demonstrated by Özakar et al.^50^. The study employed a gradient elution system comprising sodium formate, deionized water, and methanol, delivered through a 250 mm C18 column maintained at 40 °C. However, no HPTLC method has yet been established for the concurrent analysis of these three drugs, despite its notable advantages over HPLC. HPTLC enables multiple samples to be analyzed simultaneously on a single plate, enhancing analytical throughput while significantly reducing solvent consumption. Its ability to allow complete solvent evaporation prior to detection minimizes exposure to hazardous effluents and lowers environmental impact. Furthermore, HPTLC requires simpler instrumentation, making it a more cost-effective and eco-friendlier alternative for multi-component pharmaceutical analysis. Table 7 illustrates a comparison between the proposed HPTLC with other reported methods^45–50^ based on the key analytical merits and the application scope.

**Table 7:** Comparison of the proposed method with relevant reported methods.

Point of Comparison	Proposed HPTLC Method			Reported HPLC^50^			Reported HPTLC^48^		Reported HPTLC^45^		Reported HPTLC^46^	Reported HPTLC^47^	Reported HPTLC^49^
	ERD	IBU	PSE	ERD	IBU	PSE	IBU	PSE	IBU	PSE	ERD	ERD	ERD
Linearity range (µg/mL)	8-200	5-200	80-1000	5-250	5-250	5-250	45.6–75.6	6.8–11.3	500–5000	200–3000	0.5-3(µg /spot)	2.4–5.6	N/A
LOD (µg/mL)	0.014	0.002	0.150	0.10	0.25	0.10	Not reported		100.4	28.5	N/A	Not reported	N/A
LOQ (µg/mL)	0.048	0.008	0.500	0.5	1.0	0.5	Not reported		304.2	86.4	N/A	Not reported	N/A
Correlation Coefficient (r)	0.9993	0.9997	0.9995	0.9994	0.9990	0.9995	0.9934	0.9963	0.9999	0.9999	N/A	0.9993	N/A
Applications	Determine the three drugs in synthetic pharmaceutical preparations.			Evaluate the three drugs in sustained release pellet formulations.			Determine IBU and PSE in tablets.		Determine IBU and PSE in tablets.		Determine ERD in syrups.	Determine ERD in the presence of its acid degradation products.	Determine ERD in capsule and in the presence of its degradation products.

## Conclusion

This research introduces a validated and sustainable HPTLC method for the determination of ERD, IBU, and PSE simultaneously, a triple-drug combination used in the symptomatic management of URTIs. The method demonstrates excellent analytical performance, including high sensitivity, precision, and accuracy, while maintaining robustness under varied conditions. To date, no HPTLC method has been reported for this mixture, making this research a significant contribution to pharmaceutical analysis of the cited compounds. Beyond its analytical merits, the proposed method stands out for its environmental and economic sustainability. Comprehensive greenness assessments based on Analytical Eco-Scale, AGREE and GAPI approaches confirm its minimal ecological footprint, while the RGB 12 model highlights its balanced performance across analytical efficiency, environmental sustainability, and cost-effectiveness. Compared to conventional HPLC, spectrophotometric, and spectrofluorometric techniques, this HPTLC approach offers a more sustainable, high-throughput, and cost-efficient alternative, particularly appropriate for routine quality control in resource-limited and environmentally regulated settings.

Despite the promising analytical performance and eco-friendly profile of the developed HPTLC method, some limitations should be noted. The method was applied to laboratory-prepared mixtures due to the current unavailability of a marketed combined formulation containing the three drugs. Nevertheless, further application to real pharmaceutical products, once available, would be beneficial to confirm its performance in the presence of complex excipient matrices. In addition, the method was not developed as a stability-indicating one, and hence its ability to resolve the analytes from their potential degradation products under stress conditions remains unassessed. Future work may therefore focus on developing a full stability-indicating version of the method through forced degradation studies. Moreover, the separation could be further extended to include additional drugs commonly used in the treatment of URTIs, enabling broader applicability of the developed analytical platform.

## Supplementary Information

Below is the link to the electronic supplementary material.

Supplementary Material 1

## Data Availability

All data generated or analyzed during this study are included in this published article and its supplementary information file.

## References

[1] Smith, A. P. Upper respiratory tract infections and academic attainment: A case study. *J. Clin. Translational Res.* **8** (2), 156–159. 10.18053/jctres.08.202202.010 (2022). PMC9035138 35475269

[2] Virgincar, N. & Spencer, R. Diagnosis and recommended treatment of common URTIs. *Pharm. Sci. Bull.* **10** (2), 123–130 (2023). https://wchh.onlinelibrary.wiley.com/doi/pdf/10.1002/psb.244

[3] Baker, I. & Barton, E. URTIs: recommended diagnosis and treatment in general practice. *J. Gen. Pract.* **11** (1), 45–52 (2024).

[4] Kim, S. T., Park, J. S., Tae-Kim, H. & Kim, C. K. Simple determination of erdosteine in human plasma using high performance liquid chromatography. *J. Liq. Chromatogr. Relat. Technol.* **33** (13), 1319–1327. 10.1080/10826076.2010.489019 (2010).

[5] Isik, B., Bayrak, R., Akcay, A. & Sogut, S. Erdosteine against acetaminophen induced renal toxicity. *Mol. Cell. Biochem.* **287** (1), 185–191. 10.1007/s11010-005-9110-6 (2006). 16532256 10.1007/s11010-005-9110-6

[6] Madhura, V. D., Vandana, T. G. & Joshi, P. P. Dissolution method development and validation for combination of cefixime trihydrate and erdosteine capsules. *J. Pharm. Res.* **2** (11), 1700–1704 (2009).

[7] Hosoe, H. et al. Mucolytic and antitussive effects of erdosteine. *J. Pharm. Pharmacol.* **51** (8), 959–966. 10.1211/0022357991773230 (1999). 10504037 10.1211/0022357991773230

[8] Demiralay, R., Gürsan, N. & Erdem, H. The effects of erdosteine and N-acetylcysteine on apoptotic and antiapoptotic markers in pulmonary epithelial cells in sepsis. *Eurasian J. Med.* **45** (3), 167–175. 10.5152/eajm.2013.35 (2013). 25610275 10.5152/eajm.2013.35 PMC4261429

[9] Dal-Negro, R. W. Erdosteine: antitussive and anti-inflammatory effects. *Lung* **186** (1), 70–73. 10.1007/s00408-007-9065-3 (2008). 10.1007/s00408-007-9065-3 18185958

[10] Sweetman, S. C. *Martindale-The Complete Drug Reference*, 36 edn. 64, 1560, 1571 (2009).

[11] Khazaeli, P., Pardakhti, A. & Hasanzadeh, F. Formulation of ibuprofen beads by ionotropic gelation. *Iran. J. Pharm. Res.* **7** (3), 163–170. 10.22037/ijpr.2010.761 (2010).

[12] Moore, N. Ibuprofen: a journey from prescription to over-the-counter use. *J. R. Soc. Med.* **100** (48), 2–6. 10.1177/014107680710004801s01 (2007). 18335846 10.1177/014107680710004801s01

[13] Bushra, R. & Aslam, N. An overview of clinical pharmacology of Ibuprofen. *Oman Med. J.* **25** (3), 155. 10.5001/omj.2010.49 (2010). 22043330 10.5001/omj.2010.49 PMC3191627

[14] Odeku, O. A., Okunlola, A. & Lamprecht, A. Formulation and in vitro evaluation of natural gum-based microbeads for delivery of ibuprofen. *Trop. J. Pharm. Res.* **13** (10), 1577–1583. 10.4314/tjpr.v13i10.2 (2014).

[15] Głowacka, K. & Wiela-Hojeńska, A. Pseudoephedrine-benefits and risks. *Int. J. Mol. Sci.* **22** (5146), 1–11. 10.3390/ijms22105146 (2021). 10.3390/ijms22105146 PMC8152226 34067981

[16] Ahmed, M. H., Elkady, E. F., Mahmoud, S. T. & Mohamed, E. H. A green validated HPLC-UV Method for determining and quantifying pholcodine, paracetamol, and pseudoephedrine in laboratory-prepared mixtures and their FDC capsule. *Green. Anal. Chem.* **12**, 100187. 10.1016/j.greeac.2024.100187 (2025).

[17] Jain, N., Kakde, A. & Kori, M. L. Simultaneous method development for pseudoephedrine hydrochloride and desloratadine. *J. Drug Delivery Ther.* **15** (6), 6. 10.22270/jddt.v15i6.7157 (2025).

[18] Kadavilpparampu, A. M., Al-Lawati, H. A., Suliman, F. O. & Al-Kindy, S. M. Determination of the pseudoephedrine content in pharmaceutical formulations and in biological fluids using a microbore HPLC system interfaced to a microfluidic chemiluminescence detector. *Luminescence* **30** (8), 1242–1249. 10.1002/bio.2887 (2015). 25773865 10.1002/bio.2887

[19] Khan, M. M. et al. Stability indicating HPLC determination of Erdosteine in bulk drug and pharmaceutical dosage form. *J. Pharm. Biosci.* **1** (3), 105–109 (2013).

[20] Haggag, R. S. & Belal, T. S. Gradient HPLC-DAD determination of two pharmaceutical mixtures containing the antihistaminic drug ebastine. *J. Chromatogr. Sci.* **50** (10), 862–868. 10.1093/chromsci/bms082 (2012). 22677488 10.1093/chromsci/bms082

[21] Hadad, G. M., Emara, S. & Mahmoud, W. M. Development and validation of a stability-indicating RP-HPLC method for the determination of paracetamol with dantrolene or/and cetirizine and pseudoephedrine in two pharmaceutical dosage forms. *Talanta* **79** (5), 1360–1367. 10.1016/j.talanta.2009.06.003 (2009). 19635371 10.1016/j.talanta.2009.06.003

[22] Langlois, M. H., Dallet, P., Kauss, T. & Dubost, J. P. Simultaneous determination of ibuprofen and pseudoephedrine hydrochloride in pharmaceutical tablets by reversed-phase HPLC. *Anal. Lett.* **42** (18), 2951–2961. 10.1080/00032710903202006 (2009).

[23] Whelan, M. R., Ford, J. L. & Powell, M. W. Simultaneous determination of ibuprofen and hydroxypropylmethyl cellulose (HPMC) using HPLC and evaporative light scattering detection. *J. Pharm. Biomed. Anal.* **30** (4), 1355–1359. 10.1016/s0731-7085(02)00394-1 (2002). 12408926 10.1016/s0731-7085(02)00394-1

[24] Khalil, H. A., Hafez, M. G., Elgammal, F. A. H., Belal, T. S. & Gawad, D. A. Sustainable multifaceted HPLC approach for concurrent quantitation of an octa-mixture used in upper respiratory therapy with a five-dimensional sustainability assessment. *Sci. Rep.* **16** (1), 12240 (2026). 41974814 10.1038/s41598-026-45971-7 PMC13076651

[25] Prete, M. C. et al. Electrochemical determination of ibuprofen by batch-injection analysis using a BORON-doped ultrananocrystalline diamond electrode. *Electroanalysis* **37**, e202400121. 10.1002/elan.202400121 (2025).

[26] Abdoon, F. M., Salman, S. A., Hasan, H. M., Ameen, S. T. & El-Tohamy, M. F. Biogenic functionalized ZnO/CuO nanocomposite sensor for potentiometric determination of pseudoephedrine-HCl in pure and commercial products. *Baghdad Sci. J.* **21** (01), 3134–3149. 10.21123/bsj.2024.9313 (2024).

[27] Maslarska, V. Quantitative determination of some non-steroidal anti-inflammatory drugs and their acid dissociation constants by direct potentiometry. *Int. J. Appl. Pharm.* **16** (3), 320–325. 10.22159/IJAP.2024V16I3.50111 (2024).

[28] Giahi, M., Arvand, M., Mirzaei, M. & Bagherinia, M. A. Determination of pseudoephedrine hydrochloride in some pharmaceutical drugs by potentiometric membrane sensor based on pseudoephedrine–phosphotungstate ion pair. *Anal. Lett.* **42** (6), 870–880. 10.1080/00032710902722079 (2009).

[29] Jabouri, A. S. & Attia, K. F. Micro-Determination of the Macro-Drugs (Pseudoephedrine Hydrochloride-Ibuprofen) in their Pure Form and Pharmaceutical Preparations. *Macromolecular Symposia*. **414**, 2400240. 10.1002/masy.202400240 (2025).

[30] Khaki, B., Sohrabi, M. R. & Motiee, F. Development of colorimetric method for trace determination of dextromethorphan and pseudoephedrine components separately in urine matrix based on plasmonic response of gold nanoparticles: Optimization study. *Iran. J. Chem. Chem. Eng.* **43** (10), 3550–3560. 10.30492/ijcce.2024.2024169.6483 (2024).

[31] Palabiyik, I. M., Dinç, E. & Onur, F. Simultaneous spectrophotometric determination of pseudoephedrine hydrochloride and ibuprofen in a pharmaceutical preparation using ratio spectra derivative spectrophotometry and multivariate calibration techniques. *J. Pharm. Biomed. Anal.* **34** (3), 473–483. 10.1016/S0731-7085(03)00578-8 (2004). 15127802 10.1016/s0731-7085(03)00578-8

[32] Soliman, R. M. et al. Smart chemometric-assisted spectrophotometric approaches for simultaneous quantification of tertiary combination recommended for COVID-19 supportive treatments with their greenness assessment. *J. AOAC Int.* **107** (5), 774–784. 10.1093/jaoacint/qsae059 (2024). 39002112 10.1093/jaoacint/qsae059

[33] Khalil, H. A., El-Kimary, E. I., El-Yazbi, A. F. & Belal, T. S. Multiple green spectroscopic methods for erdosteine determination in bulk and dosage form with extensive greenness evaluation. *Sci. Rep.* **13** (1), 18216. 10.1038/s41598-023-45334-6 (2023). 37880475 10.1038/s41598-023-45334-6 PMC10600230

[34] Zaazaa, H. E., Elzanfaly, E. S., Soudi, A. T. & Salem, M. Y. Application of the ratio difference spectrophotometry to the determination of ibuprofen and famotidine in their combined dosage form; Comparison with previously published spectrophotometric methods. *Spectrochim. Acta Part A Mol. Biomol. Spectrosc.* **143**, 251–255. 10.1016/j.saa.2015.02.050 (2015). 10.1016/j.saa.2015.02.050 25733252

[35] Nanda, R. K., Gaikwad, J., Prakash, A., Ghosh, V. K. & Nagore, D. H. Estimation of cefixime and erdosteine in its pharmaceutical dosage form by spectrophotometric method. *Asian J. Res. Chem.* **2** (4), 404–406 (2009).

[36] Bakr, E. A., Zeid, A. M., Shalan, S. M. & El-Shabrawy, Y. Simultaneous eco-friendly spectrofluorometric estimation of itraconazole and ibuprofen in pharmaceutical and biological matrices. *Luminescence* **40**, e70071. 10.1002/bio.70071 (2025). 39746791 10.1002/bio.70071

[37] Magdy, G., Belal, F., Abdel-Megied, A. M. & Abdel-Hakiem, A. F. Two different synchronous spectrofluorimetric approaches for simultaneous determination of febuxostat and ibuprofen. *Royal Soc. Open. Sci.* **8**, 210354. 10.1098/rsos.210354 (2021). 10.1098/rsos.210354 PMC8150019 34084553

[38] Radwan, A. S., Salim, M. M. & Hammad, S. F. Synchronous spectrofluorometric methods for simultaneous determination of diphenhydramine and ibuprofen or phenylephrine in combined pharmaceutical preparations. *Luminescence* **35**, 550–560. 10.1002/bio.3750 (2020). 31904176 10.1002/bio.3750

[39] Farajzadeh, N. & Nader, N. R. A novel fluorimetric method for determination of pseudoephedrine hydrochloride in pharmaceutical formulations and blood serum. *Turk. J. Chem.* **44**, 656–669. 10.3906/kim-1912-6 (2020). 33488184 10.3906/kim-1912-6 PMC7671220

[40] El-Didamony, A. M. & Gouda, A. A. A novel spectrofluorimetric method for the assay of pseudoephedrine hydrochloride in pharmaceutical formulations via derivatization with 4-chloro-7-nitrobenzofurazan. *Luminescence* **26** (6), 510–517. 10.1002/bio.1261 (2011). 22162453 10.1002/bio.1261

[41] Damiani, P. C., Bearzotti, M. & Cabezón, M. A. Spectrofluorometric determination of ibuprofen in pharmaceutical formulations. *J. Pharm. Biomed. Anal.* **25** (3–4), 679–683. 10.1016/S0731-7085(00)00584-7 (2001). 11377049 10.1016/s0731-7085(00)00584-7

[42] Zambakjian, C. & Sakur, A. A. A new gas chromatographic method development and validation for the simultaneous determination of ibuprofen and caffeine in bulk and pharmaceutical dosage form. *Future J. Pharm. Sci.* **6** (110), 1–8. 10.1186/s43094-020-00123-0 (2020).

[43] Li, M., Guang-lei, M. & Tong-wei, Y. Determination of residual in erdosteine by headspace gas chromatography. *SA Pharm. J.* **3**, 55–57 (2013).

[44] Raj, S., Kapadia, S. & Argekar, A. Simultaneous determination of pseudoephedrine hydrochloride and diphenhydramine hydrochloride in cough syrup by gas chromatography (GC). *Talanta* **46** (1), 221–225. 10.1016/s0039-9140(97)00277-4 (1998). 18967146 10.1016/s0039-9140(97)00277-4

[45] Moustafa, A. A., Hegazy, M. A., Mohamed, D. & El-Naem, O. Simultaneous quantification of chlorpheniramine, pseudoephedrine, and ibuprofen in antitussive preparation by high-performance liquid chromatography and thin-layer chromatography–densitometric methods. *J. Planar Chromatography-Modern TLC*. **31** (4), 272–279. 10.1556/1006.2018.31.4.2 (2018).

[46] Shah, P. A., Chaudhari, D. P., Mistry, N. N. & Gandhi, T. R. Development and validation of HPTLC method for simultaneous estimation of erdosteine, guaiphenesin and terbutaline sulphate using box-behnken design. *Indian Drugs*. **54** (12), 40–50. 10.53879/id.54.12.10938 (2017).

[47] Mostafa, N. M., Badawey, A. M., Nesrine, A. T. & El-Aleem, A. B. Stability-indicating methods for the determination of erdosteine in thepresence of its acid degradation products. *J. AOAC Int.* **97** (1), 86–93. 10.5740/jaoacint.11-202 (2014). 24672863 10.5740/jaoacint.11-202

[48] Chitlange, S. S., Sakarkar, D. M., Wankhede, S. B. & Wadodkar, S. G. High performance thin layer chromatographic method for simultaneous estimation of ibuprofen and pseudoephedrine hydrochloride. *Indian J. Pharm. Sci.* **70** (3), 398–400. 10.4103/0250-474X.43018 (2008). 20046759 10.4103/0250-474X.43018 PMC2792507

[49] Mhaske, D. V. & Dhaneshwar, S. R. High-performance thin-layer chromatographic method for determination of erdosteine in pharmaceutical dosage forms. *Acta Chromatographica*. **19** (19), 170–184 (2007).

[50] Özakar, R. S. et al. A new triple combination for upper respiratory tract infections: preparation and evaluation of sustained release pellet formulations containing erdostein, ibuprofen and pseudoephedrine HCl. *FARMACIA* **70** (5), 816–830. 10.31925/farmacia.2022.5.6 (2022).

[51] Pandya, H., Devaliya, D., Shah, A. & Kotadiya, R. Chromatography chronicles: Unveiling the power of reversed-phase high-performance thin layer chromatography in pharmaceutical analysis. *Curr. Anal. Chem.* **21** (7), 777–794. 10.2174/0115734110320008240628090739 (2025).

[52] Singh, S., Khan, N., Sawant, T. & Raheja, R. Green high-performance thin-layer chromatography: A step towards eco-friendly analysis. *Chromatographia* **88** (4), 287–302. 10.1007/s10337-025-04397-5 (2025).

[53] Ahmed, H. M., Elshamy, Y. S., Talaat, W., Labib, H. F. & Belal, T. S. Simultaneous analysis of chlorzoxazone, diclofenac sodium and tramadol hydrochloride in presence of three potential impurities using validated HPLC-DAD and HPTLC methods. *Microchem. J.* **153**, 104505. 10.1016/j.microc.2019.104505 (2020).

[54] Belal, T. S., Mahrous, M. S., Abdel-Khalek, M. M., Daabees, H. G. & Khamis, M. M. Validated HPTLC method for the simultaneous determination of alfuzosin, terazosin, prazosin, doxazosin and finasteride in pharmaceutical formulations. *Anal. Chem. Res.* **1**, 23–31. 10.1016/j.ancr.2014.06.004 (2014).

[55] Imam, M. S. et al. Hasanin T.H.A. Simultaneous green TLC determination of nirmatrelvir and ritonavir in the pharmaceutical dosage form and spiked human plasma. *Sci. Rep.* **13** (1), 6165 (2023). 37061601 10.1038/s41598-023-32904-x PMC10105527

[56] ICH, Validation of Analytical Procedures: Text and Methodology, Q2(R1) (International Council for Harmonisation, 2005).

[57] Gałuszka, A., Migaszewski, Z. M., Konieczka, P. & Namieśnik, J. Analytical Eco-Scale for assessing the greenness of analytical procedures. *Trends Anal. Chem.* **37**, 61–72. 10.1016/j.trac.2012.03.013 (2012).

[58] Płotka-Wasylka, J. A new tool for the evaluation of the analytical procedure: Green Analytical Procedure Index. *Talanta* **181**, 204–209. 10.1016/j.talanta.2018.01.013 (2018). 29426502 10.1016/j.talanta.2018.01.013

[59] Pena-Pereira, F., Wojnowski, W. & Tobiszewski, M. AGREE—Analytical GREEnness metric approach and software. *Anal. Chem.* **92** (14), 10076–10082. 10.1021/acs.analchem.0c01887 (2020). 32538619 10.1021/acs.analchem.0c01887 PMC7588019

