# Supplementary material for: A novel eco-friendly HPTLC approach for the simultaneous determination of erdosteine, ibuprofen and pseudoephedrine pharmaceutical combination
Source: Sci Rep. 2026 Jun 16;16:18706. doi: 10.1038/s41598-026-57106-z (PMC13273069; doi:10.1038/s41598-026-57106-z)
Supplement: Supplementary file 1 — Supplementary Material 1 [file 41598_2026_57106_MOESM1_ESM.docx]

**Supplementary Material**

**A Novel Eco-Friendly HPTLC Approach for the Simultaneous Determination of Erdosteine, Ibuprofen and Pseudoephedrine Pharmaceutical Combination**

Feda A. H. Elgammal ^1^, Maram G. Hafez ^2^, Hadeel A. Khalil ^1^, Dina A. Gawad ^1^, Tarek S. Belal ^1^

**^1^** Pharmaceutical Analytical Chemistry Department, Faculty of Pharmacy, University of Alexandria, Elmessalah 21521, Alexandria, Egypt.

^2^ Postgraduate Student, Faculty of Pharmacy, University of Alexandria, Elmessalah 21521, Alexandria, Egypt.

Corresponding author: Tarek S. Belal

E-mail address: [tarek.belal@alexu.edu.eg](mailto:tarek.belal@alexu.edu.eg)

**Table 1S. System suitability parameters for the proposed HPTLC method**

|  | **ERD** | **IBU** | **PSE** |
| --- | --- | --- | --- |
| **R_f_ ± SD*** | 0.06 ± 0.02 | 0.23 ± 0.03 | 0.35 ± 0.02 |
| **Tailing factor (T)** | 0.86 | 1.10 | 1.25 |
| **Resolution (R_s_)** | − | 2.91 | 2.10 |

* Mean retardation factor **±** standard deviation for 5 values.

**Table 2S. Parameters of Green Analytical Procedure Index (GAPI)**

| **Proposed HPLC Method for IBU/PSE Determination**  **[22]** | **Proposed HPLC Method for ERD/IBU/PSE Determination [50]** | **Reported HPTLC Method for IBU/PSE Determination [48]** | **Reported HPTLC Method for ERD Determination [47]** | **Proposed HPTLC Method for ERD/IBU/PSE Determination** | **Parameters** |
| --- | --- | --- | --- | --- | --- |
| **Sample Preparation** | | | | | |
| At-line | | | | | **Collection (1)** |
| None | | | | | **Preservation (2)** |
| None | | | | | **Transport (3)** |
| Normal conditions | | | | | **Storage (4)** |
| Simple procedures | Direct method | Simple procedures | Simple procedures | Direct method | **Type of method:**  **direct or indirect (5)** |
| Micro-extraction | No extraction | Micro-extraction | Micro-extraction | No extraction | **Scale of extraction (6)** |
| Non-green solvents/ reagents used | Green solvents/ reagents used | Green solvents/ reagents used | Non-green solvents/ reagents used | Green solvents/ reagents used | **Solvents/reagents used (7)** |
| None | | | | | **Additional treatments (8)** |
| **Reagent and solvents** | | | | | |
| >100 mL | | 10-100 mL | | | **Amount (9)** |
| 3 | 3 | 3 | 3 | 3 | **Health hazard (10)**  **(Highest NFPA Health Score)** |
| 3 | 3 | 3 | 3 | 3 | **Safety hazard (11)**  **(Highest NFPA flammability or Instability Score)** |
| **Instrumentation** | | | | | |
| ≤1.5 kWh per sample | | | | | **Energy (12)** |
| Hermetic sealing of analytical process | | | | | **Occupational hazard (13)** |
| >10 mL | | | | | **Waste (14)** |
| No treatment | No treatment | No treatment | No treatment | Recycling | **Waste treatment (15)** |
| Yes | | | | | **Quantification** |


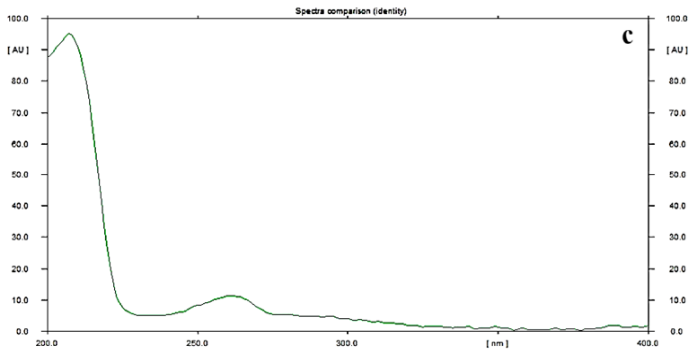

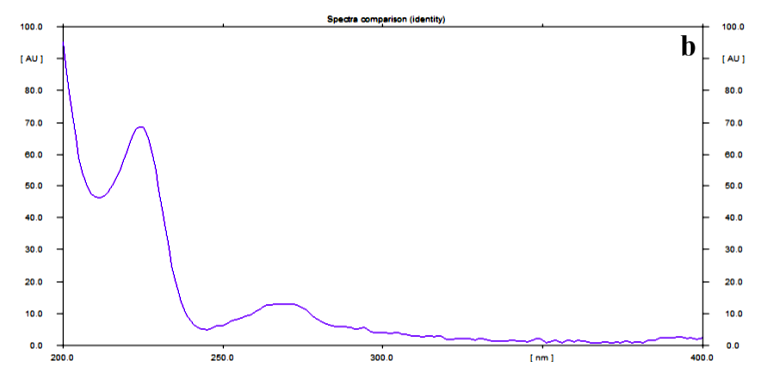

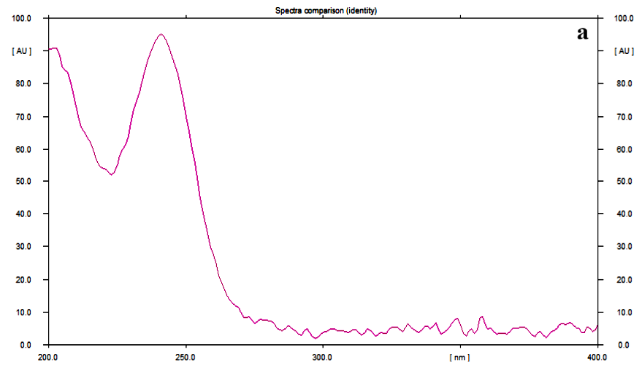


**Fig. 1S. UV-absorption spectra extracted from the HPTLC densitogram of (a) 0.5 μg/spot ERD, (b) 0.5 μg/spot IBU, and (c) 5 μg/spot PSE standard solutions.**


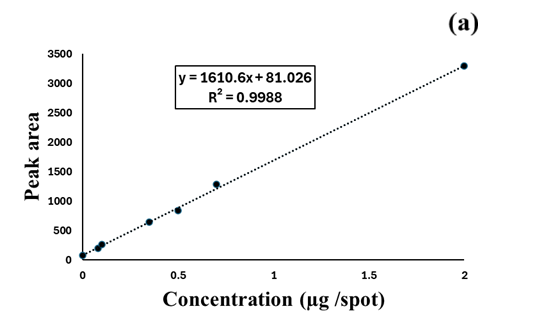

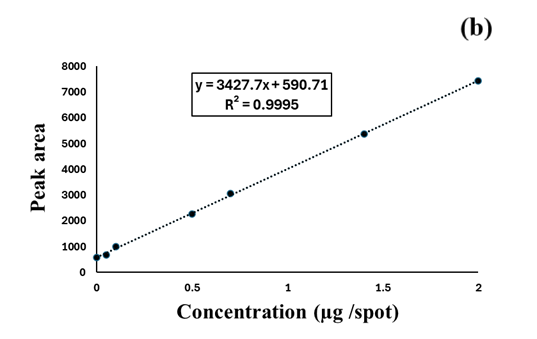

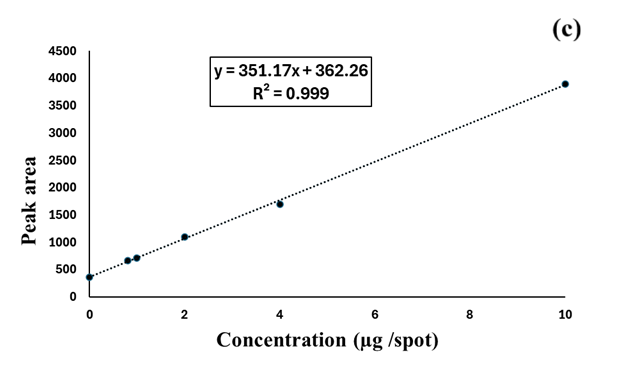


**Fig. 2S. Calibration Plots of the regression data of the peak area of (a) ERD, (b) IBU and (c) PSE at 210 nm.**


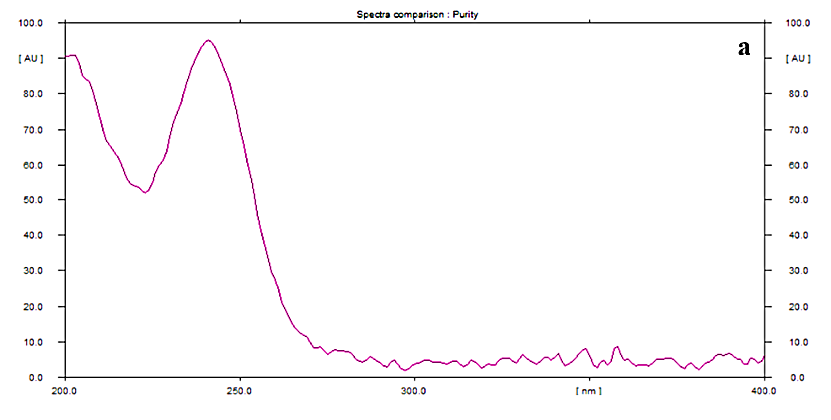

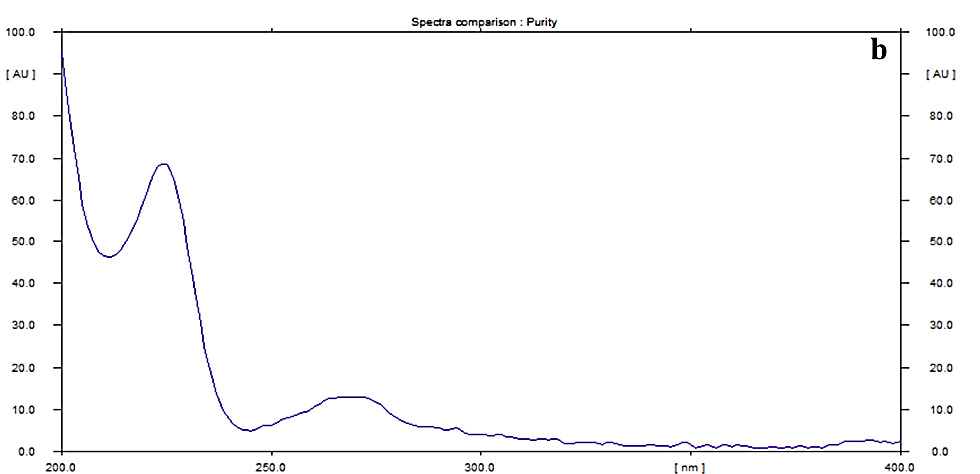

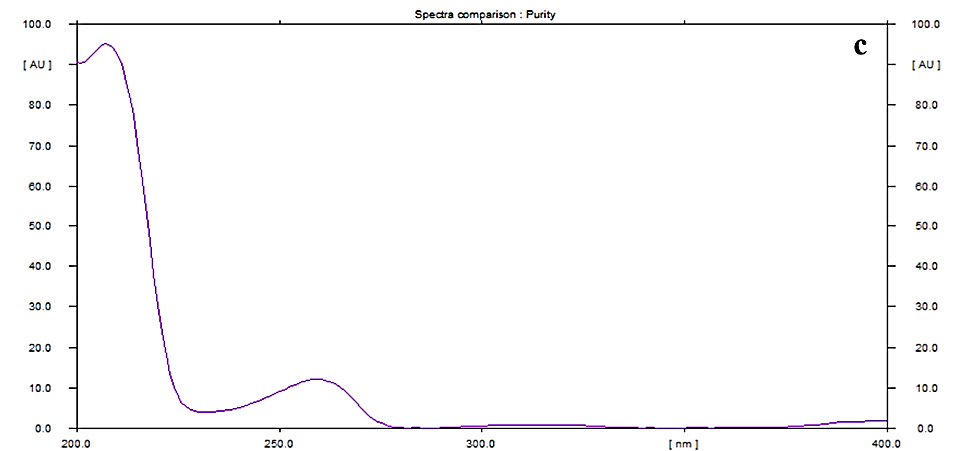


**Fig. 3S. Peak purity plots of (a) ERD, (b) IBU, (c) PSE mixture solution at R_f_ values of 0.06, 0.23 and 0.35, respectively.**
